# Combined Chronic Oral Methylphenidate and Fluoxetine Decreases D2R Levels in the Caudate Putamen and Nucleus Accumbens

**DOI:** 10.1007/s11064-025-04481-0

**Published:** 2025-07-11

**Authors:** George Lagamjis, Huy Lu, Nicole M Roeder, Brittany J Richardson, Matthew Marion, Teresa Quattrin, Lucy D. Mastrandrea, Michael Hadjiargyrou, David E. Komatsu, Panayotis K. Thanos

**Affiliations:** 1 Behavioral Neuropharmacology and Neuroimaging Laboratory on Addictions, Clinical Research Institute on Addictions, Department of Pharmacology and Toxicology, Jacob School of Medicine and Biosciences, State University of New York at Buffalo, Buffalo, NY USA; 2 UBMD Pediatrics, JR Oishei Children’s Hospital, University at Buffalo, Buffalo, NY 14203 USA; 3 Department of Biological and Chemical Sciences, New York Institute of Technology, Westbury, NY 11568 USA; 4 Department of Orthopaedics and Rehabilitation, Stony Brook University, Stony Brook, NY 11794 USA; 5 Department of Psychology, State University of New York at Buffalo, Buffalo, NY USA; 6 Department of Exercise and Nutrition Sciences, State University of New York at Buffalo, 1021 Main Street, Buffalo, NY 14203-1016 USA

**Keywords:** Psychostimulants, Serotonin Reuptake Inhibitors, Autoradiography, Reward, Brain Mapping, Dopamine

## Abstract

Methylphenidate (MP) is a commonly prescribed psychostimulant for treating Attention-Deficit/Hyperactive Disorder (ADHD). Many patients with ADHD also experience anxiety and depression, often leading to co-dosing with selective serotonin reuptake inhibitors (SSRIs), such as fluoxetine (FLX), commonly used for ADHD-related and adolescent depression. Our laboratory and others have shown that MP increases striatal dopamine (DA) transporters and DA type 1 receptor binding (D1R) in rats, and FLX has been shown to affect the DA reward pathway through the effect DA receptors play on increased cellular serotonin (5-HT). However, the effects of combined MP and FLX on DA receptor binding remain unclear. This study investigated how MP, FLX, and their combination affect D1R and DA type 2 (D2R) binding. At three weeks of age, adolescent rats received four weeks of oral drug treatments via a previously established dosing paradigm that replicates human pharmacokinetics. Rats were separated into four groups, receiving water, MP, FLX, or MP + FLX. Following treatment, autoradiography binding was conducted on coronal brain sections and showed chronic combined treatment with MP + FLX resulted in significant decreases in D2R levels relative to controls in the: Dorsal Caudate Putamen (DCPU) (51.5%), Dorsolateral Caudate Putamen (DLCPU) (50.4%), Nucleus Accumbens Core (Nac Core) (44.8%), Ventral Caudate Putamen (VCPU) (47.7%), and Ventromedial Caudate Putamen (VMCPU) (49.1%). No significant effects were reported for D1R binding. Thus, the combined treatment of MP + FLX in attenuating D2R levels may be involved in the mechanism that prior literature has described an increased risk for substance use disorder, cognitive deficits and motor dysregulation.

## Introduction

Methylphenidate (MP), marketed as Ritalin, is one of the most prescribed psychostimulants to treat attention deficit hyperactivity disorder (ADHD) in adolescents and adults [1]. As of 2022, approximately 1 in 9 school-age children have received an ADHD diagnosis, with 77.9% of those children having at least one co-occurring disorder and half of those children receiving ADHD medication [2]. These numbers have been steadily increasing in the last few decades and will only further increase as time goes on [3]. MP has also been shown to be abused recreationally by college students and adults as a cognitive enhancer [4]. The abuse and prescription of MP at these young ages presents great concern, as during these ages the brain is susceptible to developmental changes due to the changes in concentration of certain neurotransmitters and receptor levels in regions such as the prefrontal cortex, hippocampus, and limbic system. MP is a drug that can block dopamine transporters (DATs) and increase levels of extracellular dopamine (DA) in the prefrontal cortex (PFC) and the striatum. This, in turn, increases norepinephrine in the PFC and the hippocampus [5].

Fluoxetine (FLX), marketed as Prozac, is one of the most commonly prescribed selective serotonin reuptake inhibitors (SSRIs) to treat depression in adolescents and adults [6]. As of 2021, the NIH reports that five million adolescents aged 12 to 17 in the U.S. report at least one major depressive episode [7]. These numbers have been steadily increasing over the last few years and will only continue to do so as time goes on. FLX is specifically approved for the treatment of pediatric major depressive disorder (MDD) [8]. FLX is commonly prescribed in conjunction with MP to treat ADHD/MDD co-morbidity, which is shown to occur in 40% of pediatric ADHD cases [9, 10]. Alternatively, MP can also be prescribed with FLX to treat MDD alone [11]. FLX blocks the reuptake of serotonin (5-HT) in the presynaptic 5-HT neurons, which causes an increase in synaptic levels of 5-HT, and creates its anti-depressant effects [12]. The effects of FLX on the 5-HT system are well understood. However, more investigation should be done to understand the effects it may have on the dopamine (DA) reward system. SSRIs cause an increase in 5-HT levels in the presynaptic terminals; however, these elevated levels of 5-HT can be reuptake by DA transporters (DATs) into DA terminals that then release 5-HT and DA together, showing that SSRIs can indirectly affect the concentration and release of DA into the presynaptic terminal along with 5-HT [13]. These effects on DA levels are rarely ever discussed when prescribing or discussing SSRIs, and it is important to investigate and understand this relationship before prescribing these types of medications to millions of adolescents in the United States. While SSRIs have very little abuse potential, their interactions with DA levels and, subsequently, the DA reward pathway may impact people with addictive behaviors who are prescribed this drug [14–16].

The effects of MP and FLX, individually, are well understood. However, very little investigation has been conducted to understand the effects of co-dosing the two medications together. For example, it is known that FLX can increase synaptic levels of serotonin, and that MP inhibits DAT. This inhibition of DATs can cause DA to overflow, much like cocaine does [17]. Unlike cocaine, MP does not have as much affinity to serotonin transporters and does not produce serotonin overflow [17–19]. This may point to the fact that combined use of MP and FLX may induce “cocaine-like” effects by inhibiting DA and serotonin at the same time. It is important to attempt to understand the neurochemical effects of these drugs and their interactions together before prescribing them to millions of adolescents in the United States.

An important thing to note about many previous studies that look at the effects of MP and FLX on neurochemistry is that they utilize a dosing regimen that does not mimic the clinical scenario. In ADHD treatment, oral doses of 0.25–1 mg/kg MP are prescribed, resulting in plasma concentrations of 8–40 ng/mL [5]. Both MP and FLX are prescribed as oral pills. However, many animal model studies inject the drug either subcutaneously or intraperitoneally. This creates vastly different results compared to oral administration in regard to pharmacokinetic profile [20]. A major difference is also seen in the magnitude and time course of increases in extracellular DA and locomotor responses. In studies that use injection as the route of administration, doses produced plasma concentrations at the highest end of the spectrum for clinical relevance [20]. In order to maintain such high levels, an individual would have to undergo constant dosing due to the rat’s faster metabolism and shorter half-life of MP compared to humans [20]. Additionally, many of these studies last a few days, as opposed to the four-week drug treatment regimen described below. Longer treatment plans are necessary to truly understand the long-term effects of these drugs, as most cases of ADHD/MDD persist into adulthood [21].

Previously, we developed a dual-bottle eight-hour limited access drinking paradigm that allowed MP and FLX to be consumed voluntarily in the rats’ drinking water [22]. Rats were split into four groups and treated with MP, MP + FLX, FLX, and water. Drug dosages were obtained by using the rats’ previous three-day average consumption of liquid from the bottles. Rats in the MP group briefly received a dosage of 30 mg/kg for one hour and then received 60 mg/kg for the remaining seven hours. Rats in the MP + FLX group briefly received a dosage of 30 mg/kg of MP and 20 mg/kg FLX for one hour and then received 60 mg/kg of MP and 20 mg/kg of FLX for the remaining seven hours as previously described [12, 22]. Rats in the FLX group briefly received a dosage of 20 mg/kg FLX for one hour (09:00–10:00) and then received 20 mg/kg for the remaining seven hours (10:00–17:00), similar to previous studies performed by our lab [22]. The dosing of FLX was based off previous studies, which have shown to potentiate MP effects on gene expression in rats and is also consistent with human clinical FLX dosing [12]. The control group received only water for the duration of the treatment. The effect that remains most interesting is the effect of these different drug combinations on the DAergic system, and the implications it may have on abuse of these drugs and other illicit substances. This current study aims to determine the effects of combined oral MP + FLX treatment on DA type 1-like (D1R) and DA type 2-like receptor (D2R) receptor levels in relevant regions of the brain.

## Experimental Procedures

### Animals

Three-week-old Sprague Dawley rats were individually housed in humidity-controlled rooms (22 ± 2 ◦C, 50 ± 10% relative humidity) with a reverse light-dark cycle (lights off at 0800 h). Rats were split into four groups (*n* = 8–9/group): Control (drinking only water), MP, MP + FLX, and FLX. They received their respective treatment using a previously established dual-bottle eight-hour drinking paradigm [22, 23]. This paradigm allows for an MP and FLX pharmacokinetic profile that is similar to that observed in human patients with MP. With this voluntary drinking paradigm, stress is reduced compared with the gavage method.

**Fig. 1:**
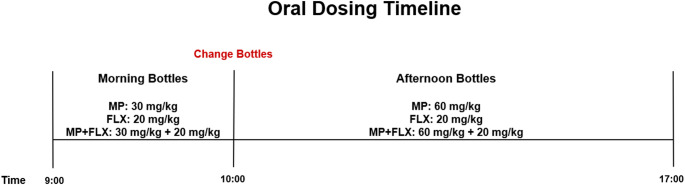
Diagram detailing dosing schedule for animals from 9:00–17:00. Rats in the MP group received a dosage of 30 mg/kg, rats in the FLX group briefly received a dosage of 20 mg/kg FLX, and rats in the MP + FLX group briefly received a dosage of 30 mg/kg of MP and 20 mg/kg FLX for one hour (9:00–10:00). For the following 7 h, rats in the MP group received a dosage of 60 mg/kg, rats in the FLX group received a dosage of 20 mg/kg, and rats in the MP + FLX group received a dosage of 30 mg/kg of MP and 20 mg/kg of FLX

Rats in the MP group briefly received a dosage of 30 mg/kg for one hour (9:00–10:00) and then received 60 mg/kg for the remaining seven hours (10:00–17:00). Rats in the MP + FLX group briefly received a dosage of 30 mg/kg of MP and 20 mg/kg FLX for one hour (9:00–10:00) and then received 60 mg/kg of MP and 20 mg/kg of FLX for the remaining seven hours (10:00–17:00) as previously described [12, 22, 24, 25]. Rats in the FLX group briefly received a dosage of 20 mg/kg FLX for one hour (9:00–10:00) and then received 20 mg/kg for the remaining seven hours (10:00–17:00) [24] (Fig. 1).

Because rats typically consume larger volumes of liquid following overnight fluid restriction, a lower dose of MP is given in the first bottle while still bringing the plasma concentrations into the target range. During the remaining seven hours, rats will drink smaller volumes intermittently, the higher dose during this time produces the target plasma concentration range. Rats underwent this treatment every day for four weeks. Following the treatment period, all the rats in each group were euthanized with isoflurane (3.0%) and brains were extracted, flash frozen, and stored at -80 C. All experiments were approved by the University at Buffalo Institutional Animal Care and Use Committee.

### Drugs

MP hydrochloride was dissolved in distilled water to produce 30 and 60 mg/kg solutions. FLX was dissolved in distilled water to produce 20 mg/kg solutions (Sigma Aldrich, St. Louis, MO). Bottles were made fresh every day based on body weight and average fluid consumed from the last three days to calculate dosages.

### D1R Autoradiography

D1R binding was evaluated using [³H] SCH 23390 autoradiography. Binding was performed as previously described [5, 26–30]. Slides were preincubated for 60 min at room temperature in 50 nM Tris HCl buffer (120 mM NaCl, 5 mM KCl, 2mM CaCl2, 1 mM MgCl2, pH = 7.4). Afterward, 2.5 nM [³H] SCH 23390 (specific activity = 85 Ci/mmol and 40 nM ketanserin were added to the pre-incubation buffer followed by an additional 60 min of incubation at room temperature. Non-specific binding was determined in the presence of 1 µM flupenthixol. Slides were then washed twice for 5 min at 4^0^C in pre-incubation buffer followed by a brief immersion at 4^0^C in dH2O.

### D2R Autoradiography

D2R binding was determined using [³H] spiperone autoradiography. Binding was performed as previously described [5, 26–30]. Slides were preincubated for 60 min at room temperature in 50 mM Tris HCl buffer (120 mM NaCl, 5 mM KCl, 2 mM CaCl2, 1 mM MgCl2, pH = 7.4). Afterward, 0.5 nM [³H] spiperone (specific activity = 16.2 Ci/mmol) and 40 nM ketanserin were added to the pre-incubation buffer followed by another 60-minute incubation at room temperature. Non-specific binding was determined in the presence of 10 µM sulpride. Slides were then washed twice for 5 min at 4^0^C in pre-incubation buffer followed by brief immersion at 4^0^C in dH2O.

### Regions of Interest (ROI) Analysis

Once the slides dried, they were opposed to BioMaxXAR film (D1: 6 weeks, D2: 10 weeks) with tritium standards in light-sensitive cassettes. [³H] SCH 23390 and [³H] spiperone binding density was measured using ImageJ software. Regions of interest include the Dorsal Caudate Putamen (DCPU), Dorsolateral Caudate Putamen (DLCPU), Dorsomedial Caudate Putamen (DMCPU), Ventral Caudate Putamen (VCPU), Ventrolateral Caudate Putamen (VLCPU), Ventromedial Caudate Putamen (VMCPU), Nucleus Accumbens Core (Nac Core), Nucleus Accumbens Shell (Nac Shell), the Substantia Nigra (SNR) and the Olfactory Tubule (OT). Note that DCPU and VCPU are not the combinations of the lateral and medial CPUs, but rather a region independent of those. Specific binding was calculated by subtracting the nonspecific binding from the total binding and is expressed in µCi/g tissue. Damaged tissues were excluded from the study.

### Statistics

Specific [³H] SCH23390 and [³H] spiperone binding for each ROI was analyzed using one-way ANOVAs following the four-week treatment of water, MP, MP + FLX, and FLX groups. All statistical analyses and graphing were performed using GraphPad Prism 10, with statistical significance set at a = 0.05. Data was tested for normality before determining significance. Tukey’s HSD was performed for all significant main effects. Values are expressed as a total [³H] SCH 23390 and [³H] spiperone binding means (µCi/g) ± S.E.M for their respective receptors.

## Results

### [³H] SCH 23,390 Autoradiography

**Fig. 2:**
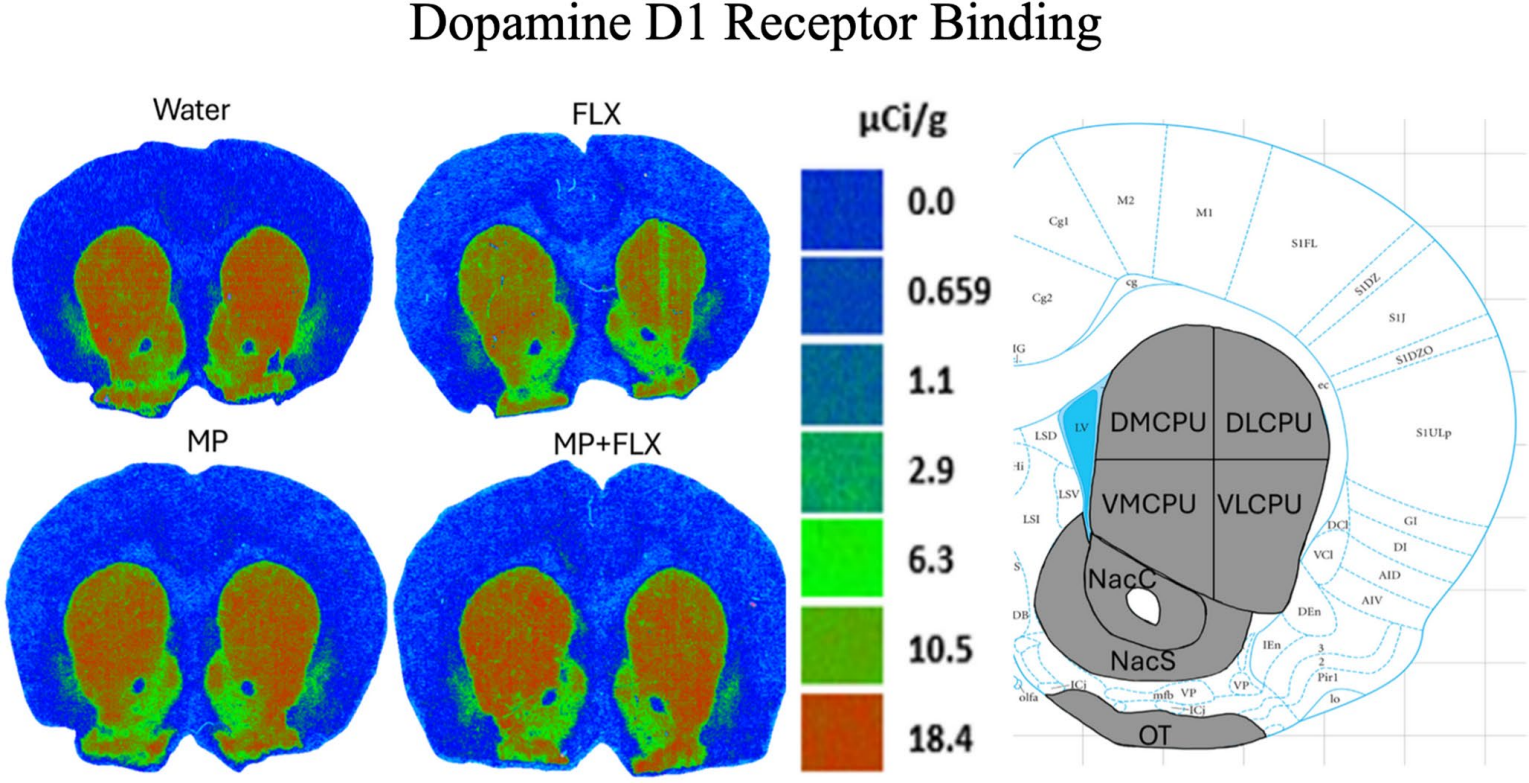
Representative figures showing D1R binding using [³H] SCH23390 to assess binding levels, with attached atlas figure showing: Dorsomedial Caudate Putamen (DMCPU), Dorsolateral Caudate Putamen (DLCPU), Ventromedial Caudate Putamen (VMCPU), Ventromedial Caudate Putamen (VMCPU), Ventrolateral Caudate Putamen (VLCPU), Nucleus Accumbens Core (NacC), Nucleus Accumbens Shell (NacS), and Olfactory Tubercle (OT)

**Fig. 3:**
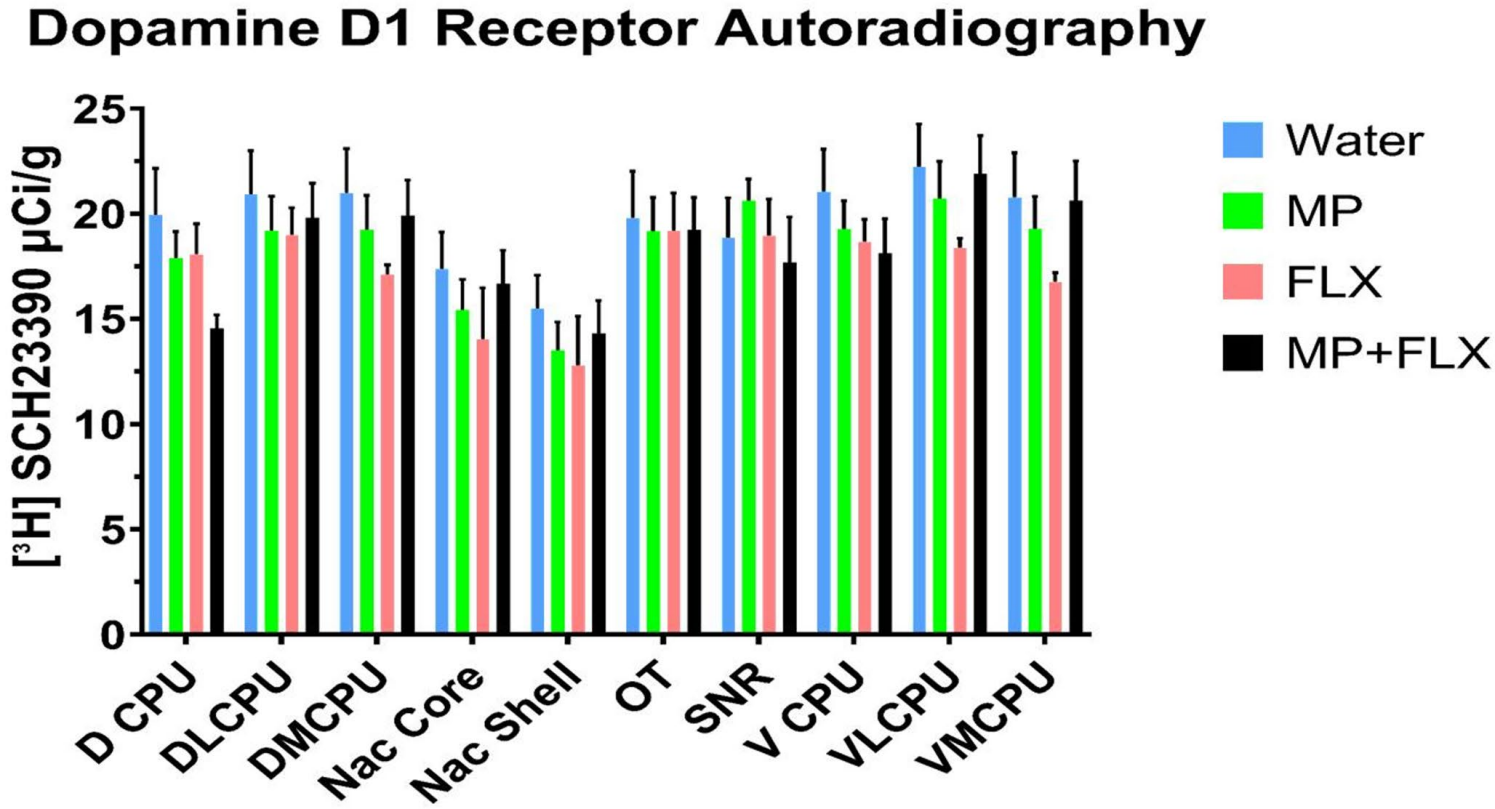
Mean [³H] SCH 23,390 binding (µCi/g) ± SEM in the Basal Ganglia following 4 weeks of treatment, with *n* = 8/9 per group. Quantitative autoradiography of [³H] 23,390 (D1R-like) binding levels within the Dorsal Caudate putamen (D CPU), Dorsolateral Caudate Putamen (DL CPU), Dorsomedial Caudate Putamen (DM CPU), Nucleus Accumbens Core (Nac Core), Nucleus Accumbens Shell (Nac Sh Shell), Olfactory Tubercle (OT), Substantia Nigra (SNR), Ventral Caudate Putamen (V CPU), Ventrolateral Caudate Putamen (VL CPU), and the Ventromedial Caudate Putamen (VM CPU) across all drug treatment groups. Measurements of the regions of interest were carried out at the bregma coordinates taken from the Paxinos & Watson rat brain atlas. No significant difference was observed (*p* > 0.05) across any of the groups. Each bar represents the group mean for dopamine D1 receptor binding

D1R specific binding was assessed using [³H] SCH 23390 after four weeks of drug treatment and was analyzed with a one-way ANOVA, with drug treatments as a factor (Water, MP, MP + FLX, FLX) in the basal ganglia. This one-way ANOVA and Tukey’s Post hoc were used to test for significance, and no significant effects were found across all four groups for [³H] SCH 23390 (D1R) binding. Specifically, in the D CPU [F(3,30) = 1.834; *p* > 0.05], DL CPU [F(3,32) = 0.2613; *p* > 0.05], DM CPU [F(3,30) = 0.8561, *p* > 0.05], V CPU [F(3, 32) = 0.6657, *p* > 0.05), VL CPU [F (3, 30) = 0.9187; *p* > 0.05], VM CPU [F (3, 30) = 1.062; *p* > 0.05], Nac Core [F (3, 32) = 0.6346, *p* > 0.05], Nac Shell [F (3, 32) = 0.4327; *p* > 0.05], OT [F (3, 32) = 0.03039; *p* > 0.05], and SNR [F (3, 28) = 0.4250; *p* > 0.05]. A one-way ANOVA found no significant interactions or main effects across all four groups (*p* > 0.05; Figs. 2 and 3).

### [³H] Spiperone Autoradiography

**Fig. 4:**
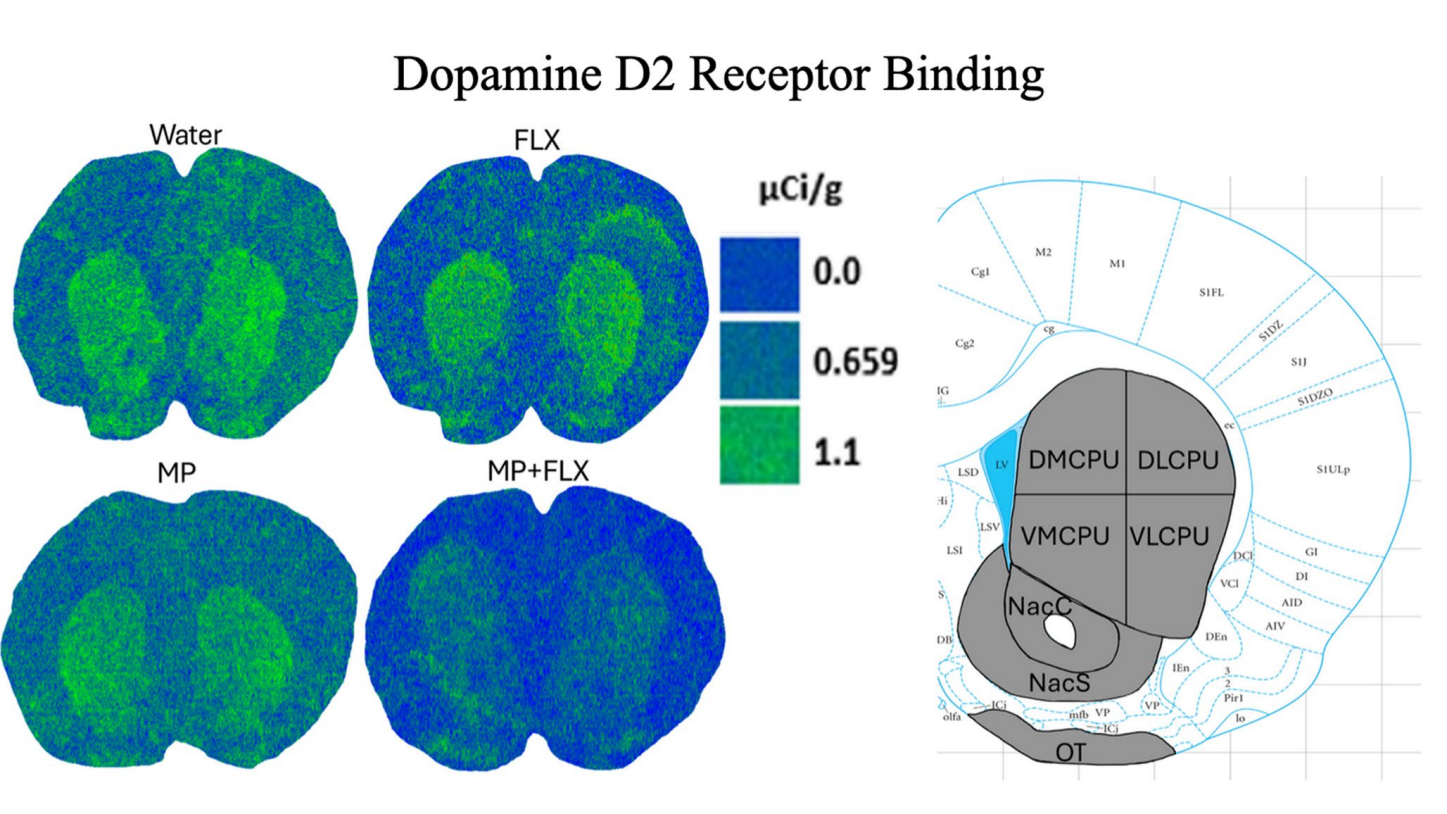
Representative figures showing D2R binding using [³H] spiperone to assess binding levels with attached atlas figure showing: Dorsomedial Caudate Putamen (DMCPU), Dorsolateral Caudate Putamen (DLCPU), Ventromedial Caudate Putamen (VMCPU), Ventrolateral Caudate Putamen (VLCPU), Nucleus Accumbens Core (NacC), Nucleus Accumbens Shell (NacS), and Olfactory Tubercle (OT)

**Fig. 5:**
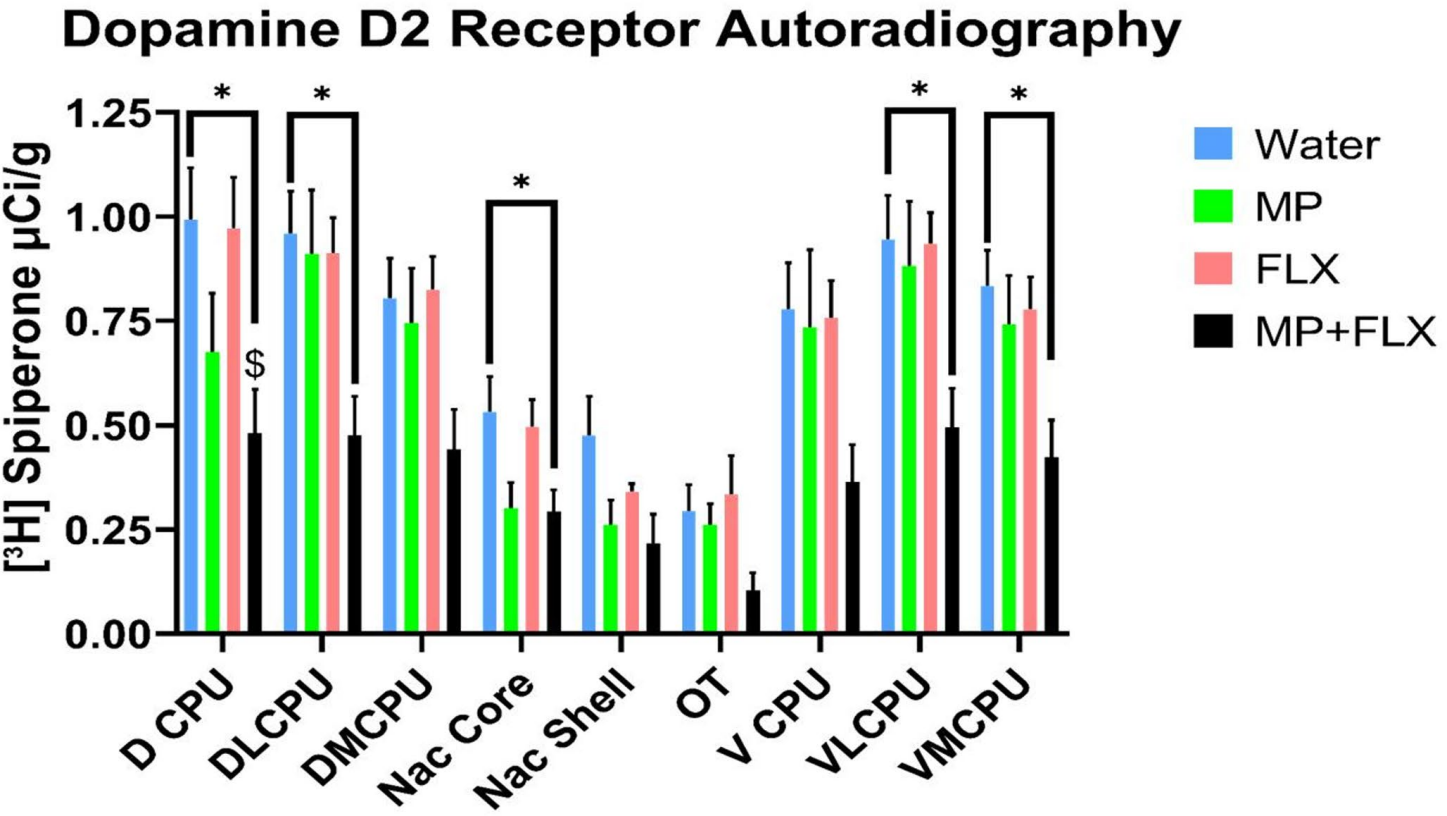
Mean [³H] spiperone binding (µCi/g) ± SEM in the Basal Ganglia following 4 weeks of treatment, with *n* = 8/9 per group. * Denotes a significant difference (*p* < 0.05) between MP + FLX and Water. $ denotes a significant difference (*p*< 0.05) between MP + FLX and FLX. ROIs consisted of the Dorsal Caudate putamen (D CPU), Dorsolateral Caudate Putamen (DL CPU), Dorsomedial Caudate Putamen (DM CPU), Nucleus Accumbens Core (Nac Core), Nucleus Accumbens Shell (Nac Shell), Olfactory Tubercle (OT), Ventral Caudate Putamen (V CPU), Ventrolateral Caudate Putamen (VL CPU), and the Ventromedial Caudate Putamen (VM CPU)

D2R specific binding was assessed using [³H] spiperone after four weeks of drug treatment and was analyzed with a one-way ANOVA, with drug treatments as a factor (Water, MP, MP + FLX, FLX) in the basal ganglia (*p* < 0.05; Figs. 4 and 5; Table 1). A significant decrease in D2R binding in the MP + FLX group was observed using Tukey’s Post hoc for the D CPU [F (3, 24) = 3.847; *p* < 0.05], DL CPU [F (3, 26) = 3.794; *p* < 0.05], Nac Core [F (3, 24) = 3.373; *p* < 0.05], VL CPU [F (3, 26) = 3.370; *p* < 0.05], VM CPU [F (3, 25) = 3.708; *p* < 0.05]. Tukey’s post hoc test found that, compared to water, MP + FLX-treated rats were shown to have less [³H] D2R binding in the listed regions. Additionally, compared to the FLX group, MP + FLX-treated rats were shown to have less D2R binding in the DCPU. No significance was observed across all other brain regions and specifically in the DM CPU [F (3, 26) = 2.780; *p* > 0.05], Nac Shell [F (3, 19) = 2.781; *p* > 0.05], OT [F (3, 13) = 1.104; *p* > 0.05], V CPU [F (3, 26) = 2.191; *p* > 0.05].

**Table 1:** Table summarizing the percent differences in average binding between the water and MP + FLX groups in regions of significance for D2R mean [³H] spiperone binding (µCi/g). ROIs consist of the dorsal caudate putamen (D CPU), dorsolateral caudate putamen (DL CPU), nucleus accumbens core (Nac core), Ventrolateral caudate putamen (VL CPU), and the Ventromedial caudate putamen (VM CPU). All percents indicate a percent decrease in mean binding values

Dopamine D2 Receptor Water vs. MP + FLX Summary			
Region of Interest	Water (Mean Binding µCi/g)	FLX (Mean Binding µCi/g)	% Difference
D CPU	0.9933	0.4816	51.5%
DL CPU	0.9601	0.4763	50.4%
Nac Core	0.5323	0.2936	44.8%
VL CPU	0.9457	0.4944	47.7%
VM CPU	0.8342	0.4245	49.1%

## Discussion

This study aimed to examine the effects of independent dosing and co-dosing of methylphenidate and fluoxetine on D1R and D2R binding. Drug treatment began at three weeks of age when major development of the DAergic reward system takes place [31] and is more sensitive to changes at this time. Notably, a dual bottle drinking paradigm was used to administer the medication. This paradigm allows for an MP pharmacokinetic profile that is similar to that observed in human patients taking oral MP. Additionally, the length of treatment of 4 weeks was chosen based on the proportionality of a rat’s lifespan to a human. For example, 13.8 days is a rat’s life translates to approximately 1 year for a human’s life [32]. This would mean that 4 weeks in a rat’s life would translate to about 2 years for a human. This fact was taken into consideration when designing the dosing paradigm, given that humans take these drugs for months, or even years. Results of the current study show significant binding effects on D2R binding in the D CPU, DL CPU, Nac Core, VL CPU, and VM CPU between the water the MP + FLX-treated rats, with MP + FLX showing less D2R binding levels in the regions listed. No significant effects on D1R binding were found.

D1R and D2R are involved in locomotion, learning and memory, attention, impulse control, and sleep [33]. Dysfunctions of these behaviors and processes are present in patients who suffer from ADHD and depression and are treated by a dual prescription of MP + FLX [11]. D1R is classified as excitatory, while D2R is inhibitory. These receptors work in tandem with the ventral tegmental area (VTA) of the brain [34, 35]. The VTA is a brain structure that serves a central role in motivation, reward processing, cognition, locomotion, as well as different sexual behaviors [36, 37]. The VTA also plays a pivotal role in the mesolimbic reward pathway, which is responsible for most of the characteristics of addiction [38]. When the feeling of reward is experienced, the mesolimbic system is activated and releases DA into targeted nuclei. The ventral striatum, including the nucleus accumbens, is a major substrate involved in reward [38]. Previous studies have shown that MP and FLX co-dosing have a significant effect on gene expression that mimics cocaine and other addiction-related gene regulation, with zif-268 and c-fos [39, 40].

MP acts as a DA reuptake inhibitor, increasing DA in the presynaptic cleft. FLX acts as an SSRI, increasing 5-HT in the presynaptic cleft. When dosed alone, no effect was shown on either D1R or D2R binding; however, when combined, there was a significant decrease in D2R binding. Very little research has been conducted investigating the effects of combining these two drugs. Previous studies in our lab have detailed the effects of MP on D1R and D2R binding [12, 24]. Other studies have also detailed the effects FLX has on MP drug actions [12, 39]. D2R activity, especially in the regions affected, has been shown to play a key role in learning and memory, as well as acquiring addictive behaviors [41]. Decreasing D2R in these regions can impact these behaviors. Additionally, decreases in overall D2R levels have been seen in individuals with substance abuse disorder [42]. The combination of FLX and MP has also been shown to significantly affect behavior as well as gene expression in other works. It has been hypothesized that the combined effects of decreasing both DA and 5-HT can explain the effects seen [12, 25].

With this voluntary drinking paradigm, stress would be reduced compared to the gavage method. Other studies have used IP injections to administer medication, which can return different and inaccurate results relating to a human patient [43]. Exclusively male rats were used in this study. Since there are known sex differences in the DAergic system [44], future studies should aim to include both sexes in the experiment or aim to examine the effects on female rats specifically. Our previous studies primarily looked at MP alone at different dosages while this study examined multiple groups with different dosages of MP and the addition of FLX alone and combined with MP [5, 22].

A key difference between this study and one previously conducted by our lab is the significance found in D1R in the MP group. In this study, no significance was found in any region with D1R binding, yet in previous studies, we had found a significant decrease in D1R binding in multiple regions of the brain [5]. This difference can be explained by the different methodologies. The present study treated the rats for four weeks, while the prior study treated for much longer (thirteen weeks). This brings up the point that longer durations of treatment could yield even greater effects on the DAergic system.

Zif-268 and c-fos are both related to memory formation, learning, neuroplasticity, and motivation, and a lack of either of these can result in the impairment of these functions [45, 46]. Specifically, zif-268 is a transcription factor that measures the transition from short to long-term synaptic plasticity and the expression of long-term memories and plays a role in hippocampal long-term potentiation [47]. C-fos is a marker used for neuronal activity throughout the brain and helps map out neuronal circuits that function in behavioral responses induced by stress [48, 49]. Other studies have shown that D2R completely suppresses basal levels of zif-268 and marginally increases levels of c-fos, while D1R has been shown to enhance levels of zif-268 and c-fos [50–52]. This action of suppression and stimulation comes from the fact that D2 receptors inhibit gene expression [52–54]. However, a decrease in these D2 neurons increases gene expression in striatal neurons, with the effect presumably coming from the fact that D2R inhibit second messenger signal, opposing the stimulatory action of D1 [52]. Yet, potentiation of gene regulation in striatonigral neurons comes from the combined stimulation of D2 and D1 receptors, an interaction that is thought to be mediated by cholinergic interneurons [52, 55–57]. Interestingly, FLX has been shown to potentiate MP-induced c-fos expression in different areas of the striatum [39]. Given that the presence of D2 decreases levels of zif-268 and c-fos and that FLX potentiates MP-induced c-fos expression in different areas of the striatum, we should see that the decrease in D2R binding levels caused by MP + FLX in specific regions of the brain should decrease levels of c-fos in the brain. The data presented confirms findings from previous studies showing the effects of MP and FLX on zif-268 and c-fos gene expression [39].

The caudate putamen (CPU) plays an essential role in learning and motor control, speech articulation, language functions, reward, cognitive functioning, and addiction [58]. In this experiment, a significant decrease in D2R was found in multiple regions of the CPU between MP + FLX and water. These regions include the D CPU, which is involved in initiating learning and habit formation [59], the DLCPU, which is involved in the later phases of acquiring addictive behavior [60], and the VLCPU and VMCPU, which are both involved in processing and regulating motor outputs and expressing reward signals during motivated behaviors [61]. These regions all demonstrated lower levels of D2R binding compared to the control group. CPU dysfunction, as well as changes in DA levels, primarily decreases in DA levels in the caudate putamen, have been shown to be related to Parkinson’s disease, Huntington’s disease, Alzheimer’s disease, depression, obsession-compulsive disorder, Wilson disease, and autism [58]. Another key function of the CPU is its role in reversal learning [62]. Reversal learning refers to the ability to actively suppress reward-related responses and to cease current behavior [63, 64]. Both D1R and D2R modulate different stages of reversal learning, with a decrease in D2R impairing the ability to stop current, reward-seeking behavior [62, 65]. This implies that, in lowering D2R levels in the CPU, MP + FLX could impair the reversal learning behavior, making it difficult for patients to cease reward-seeking behaviors, such as drug abuse and misuse. DA in the CPU, particularly the dorsal section, also plays a major role in motor planning and execution [66]. A decrease in these DA levels can lead to motor dysfunction, similar to that seen in patients with Parkinson’s disease.

The nucleus accumbens (Nac) is responsible for many different functions in the brain but primarily being considered the neural interface between motivation and action. It also plays a role in feeding behaviors and drug-related reward behaviors [67]. The Nac is split up into two sections: the core and the shell. The shell acts as a ‘coincidence detector’, which can be activated during adaptive behavioral situations, which is then mediated by the core. The core and the shell work in tandem with each other, and DA released in that region acts as a stabilizer for its processes [67]. Our previous studies and others have looked at the effects of these drugs on the reward pathway as well as the type of behavior exhibited by these medications; as such, the Nac remains a very important region of interest across our studies.

In this study, we saw a significant effect in D2R binding in the Nac Core, with the MP + FLX having less overall binding than the water group in the Nac Core. Nac DA has been shown to be essential to promote the behavioral response to reward-predictive cues [41]. A decrease in overall D2R can lead to a great impact on this and many other behaviors. Activation of D2R has an inhibitory effect on neurons in the Nac [36]. D2R dysregulation, such as a decrease in D2R, is often seen in individuals with substance abuse disorder [42].

### Limitations and Future Directions

This study focuses on the combined and independent treatment of MP and FLX, by comparing treatment groups of MP, FLX, MP + FLX, or water. Future research will examine other relevant SSRIs and their effects on dopamine signaling. While this study assessed male rats, future investigation will be needed on females. Additionally, future studies could investigate the pharmacokinetics of these drugs following chronic oral treatment. Other limitations when comparing the present study with prior papers were the difference in what brain regions were outlined and used to quantify D1R and D2R as well as different film types, and development times. Future research will examine the effects of other SSRIs along with pharmacokinetics of these drugs when administered orally, chronically, and in combination. Additionally, future research will assess how D2R activity affects transcription factors such as Zif-268 and c-fos.

## Conclusion

This study described the effects of chronic (4 weeks) combined oral use of the psychostimulant MP and the SSRI FLX on D1R and D2R binding levels in the brain. While there was no significant effect found on D1R, there was a significant decrease in D2R found in all subregions of the caudate putamen (D CPU, DL CPU, VL CPU, and VM CPU) and the nucleus accumbens. D2R plays important roles in learning, memory, attention, sleep, and reward seeking behavior. Prior research has documented that decreases in D2R levels of the brain have been associated with disruptions in these behaviors. Finally, these results help explain the mechanism of recent research showing that the same combined oral treatment of MP + FLX resulted in increased risk for drug abuse (Senior et al. 2023).

## Data Availability

No datasets were generated or analysed during the current study.
